# Clinical Expertise Is Core to an Evidence-Based Approach to Auditory Processing Disorder: A Reply to Neijenhuis et al. 2019

**DOI:** 10.3389/fneur.2019.01096

**Published:** 2019-10-18

**Authors:** Vasiliki Iliadou, Christiane Kiese-Himmel, Doris-Eva Bamiou, Helen Grech, Martin Ptok, Gail D. Chermak, Hung Thai-Van, Tone Stokkereit Mattsson, Frank E. Musiek

**Affiliations:** ^1^Clinical Psychoacoustics Lab, Third Department of Psychiatry, Neuroscience Sector, Medical School, Aristotle University of Thessaloniki, Thessaloniki, Greece; ^2^Phoniatric and Pediatric Audiological Psychology, University Medical Center Göttingen, Georg-August-University, Göttingen, Germany; ^3^Faculty of Brain Sciences, University College London Ear Institute, University College London, London, United Kingdom; ^4^Department of Communication Therapy, Faculty of Health Sciences, University of Malta, Msida, Malta; ^5^Department of Phoniatrics and Pediatric Audiology, Medizinische Hochschule Hannover, Hanover, Germany; ^6^Department of Speech and Hearing Sciences, Elson S. Floyd College of Medicine, Washington State University Health Sciences Spokane, Spokane, WA, United States; ^7^Department of Audiology and Otoneurological Evaluation, Hospices Civils de Lyon, Paris Hearing Institute, Centre de l'Institut Pasteur, Inserm U1120, Paris, France; ^8^Department of Otorhinolaryngology, Head and Neck Surgery, Ålesund Hospital, Ålesund, Norway; ^9^Neuroaudiology Lab, University of Arizona, Tucson, AZ, United States

**Keywords:** auditory processing disorder, evidence-based approach, hearing, pediatrics, systematic reviews

## Abstract

The opinion article “An Evidence-based Perspective on Misconceptions Regarding Pediatric Auditory Processing Disorder” by Neijenhuis et al. (1) presents a distorted view of the evidence-based approach used in medicine. The authors focus on the amorphous non-diagnostic entity “listening difficulties” not auditory processing disorder (APD) and create confusion that could jeopardize clinical services to individuals with APD. In our perspective article, we rebut Neijenhuis et al. (1), and more importantly, we present a rationale for evidence-based practice founded on the premise that research on APD is only clinically applicable when conducted on clinical populations diagnosed with APD.

## What is an Evidence-based Approach to Pediatric APD?

The answer is that it is the same as for any other disease or disorder. According to Sackett et al. (2), the evidence-based approach in medicine includes three elements: (a) clinical expertise, (b) best research available, and (c) the patient's values and preferences. While recognizing the essential role of research in establishing the evidence-base for a disorder, one must not discount the other two pillars. While some might be tempted to prioritize research (even research with methodological and/or design faults) over clinical expertise in one's conceptualization of evidence-based practice, Haynes et al. (3) place clinical expertise at the *core* of the clinical decision process. A key element for an evidence-based approach to APD is that the approach is informed by research conducted on clinical populations *diagnosed* with APD (4).

In their opinion paper, Neijenhuis et al. (1) assert that the three “systematic reviews” they cite undermine the evidence-base for APD. In fact, none of these papers is actually a systematic review of primary, peer-reviewed research conducted with participants diagnosed with APD. In the absence of published systematic reviews of participants diagnosed with APD at the present time, the next best evidence-based step is to use current professional association guidelines. European guidelines (5) refer to many countries' guidelines (within and beyond Europe) and these provide analogous and consistent approaches to the diagnosis of APD. This approach is not analogous to the approaches taken in the papers cited as systematic APD reviews by Neijenhuis et al. (1). In fact, they cite a review of clinical practice guidelines (6), in which the authors employed an appraisal method for guidelines using the Agree II tool to rate each guideline's scientific approach. Heine and O'Halloran concluded that all available APD guidelines in the English language (including the American and British Guidelines) scored low in most domains primarily due to “poor methodological reporting” and should not be used in their current form. It should be noted that a systematic search and evaluation of clinical practice guidelines is not a systematic review of primacy research, and, moreover, is limited to clinical trials (7).

The second alleged systematic review cited (8) included research involving participants “suspected of” rather than diagnosed with APD. The third paper they characterized as a systematic review (9) is in fact a report of research in which comparisons were made between a clinical group referred for APD evaluation (but not diagnosed with APD) and a control group of children. The authors reported correlations between auditory processing scores and cognitive scores, concluding that cognitive testing is essential in APD diagnosis. This is, simply put, illogical. Finding correlations between these two variables in undiagnosed participants tells us little about the use of cognitive measures in a test battery designed to diagnose APD. Any conclusions made by reviews of APD that include children suspected of APD or diagnosed with APD on the basis of self-report or informant's report on questionnaires or based on children with general listening difficulties do not provide the best available evidence elucidating APD. Drawing conclusions based on performance of poorly defined participants poses significant threats to the validity of the research. Results of any study that uses the metric “*suspected of APD*” or “listening difficulties” cannot be relied upon because: (i) One cannot be sure whether the participants in the study presented any type of *true* auditory deficit, and (ii) the participants may have had a wide range of unidentified issues (10). Efficient (i.e., sensitive and specific) clinical tests of auditory processing must be used to clearly define participants and to identify and describe known comorbidities so that analyses can be conducted and results interpreted accurately (11). The serious limitations of the *suspected of APD* label (that is not a diagnosis) is corroborated by the finding that many children referred for central auditory processing evaluations because of “listening difficulties” actually perform quite well on central auditory processing measures (12, 13). One would assume that if parent and teacher reports were good predictors of auditory processing difficulties, then the APD hit rate (i.e., true positives) for these referrals would be much higher. The reality is that there are no published, true systematic reviews of appropriately diagnosed individuals with APD. Labeling children suspected APD rather than evaluating and appropriately diagnosing for APD is not evidence-based and is threatening the intervention services provided to APD diagnosed individuals.

The European Consensus APD Clinical Practice Guidelines (5) are predicated on research similar to that underlying the ASHA and AAA Guidelines (14, 15). Neijenhuis et al. (1) attempt to undermine confidence in current guidelines without presenting any alternative, evidence-based approach for the diagnosis of APD. Moreover, they incorrectly assert that APD may be better explained by other developmental disorders, including attention or language-listening deficits. This is an assumption/argument that published research has failed to confirm. Published research shows that a small sub-group of APD diagnosed children present co-morbid attention deficits (16). Moreover, abnormal performance on auditory processing tests often occurs despite sustained attention within normal limits (17). In fact, most auditory processing tests share only a mild to moderate degree of variance with cognition, leading Weihing et al. (18) to conclude that auditory processing performance is not driven by cognition alone. In addition, maturation rates for different auditory tasks are not correlated, as would be expected if a non-sensory factor (e.g., attention) had a uniform influence on performance (19–21). Would Neijenhuis et al. (1) assert that a poor response to an auditory stimulus (in the presence of normal hearing sensitivity) is due to poor attention, but a poor response to a visual stimulus must be due to a visual processing deficit (in the presence of normal peripheral vision)? Grounds to argue either case are lacking. Recent research also shows that although attention is correlated with how well or poorly a *typically developing child* uses rhythm to perceive speech in babble, this is not true for APD diagnosed children (22). In fact, not surprisingly, there is evidence to suggest that APD (as well as peripheral hearing impairment) can affect measures of cognition (23, 24). Clearly, brain organization and processing underlies bidirectional interactions, as well as comorbidity (25).

## What is the APD Clinical Reality in Europe and USA?

APD is diagnosed by appropriately educated otorhinolaryngologists (ENTs) and/or audiologists since few would dispute that an APD assessment begins with a thorough assessment of “peripheral” hearing function. The diagnosing clinician needs to have an in depth understanding of auditory function and related pathologies, so ENTs/audiologists need to be key members of any multidisciplinary team, where such health care personnel are available. However, we must not lose sight of the limited resources in different countries, in which a multidisciplinary approach is advocated, but may not be possible within a formal team setting. Differential diagnosis requires multidisciplinary assessment involving speech-language pathologists, educators, and psychologists (26, 27). This comprehensive evaluation is best practice in APD diagnosis, as well as in formulating individualized intervention. In all cases, research should guide practice, provided that the research is based on the intended population of clinical interest—that is, individuals diagnosed with APD. Auditory processing tests are evaluated for their sensitivity and specificity before they are used for clinical diagnosis (27–30). APD clinical diagnosis reveals the presence of heterogeneity in the specific auditory processing deficits seen in individuals diagnosed with APD. Thus, there is a documented need for further research in this domain implemented in APD diagnosed individuals.

**Figure 1:**
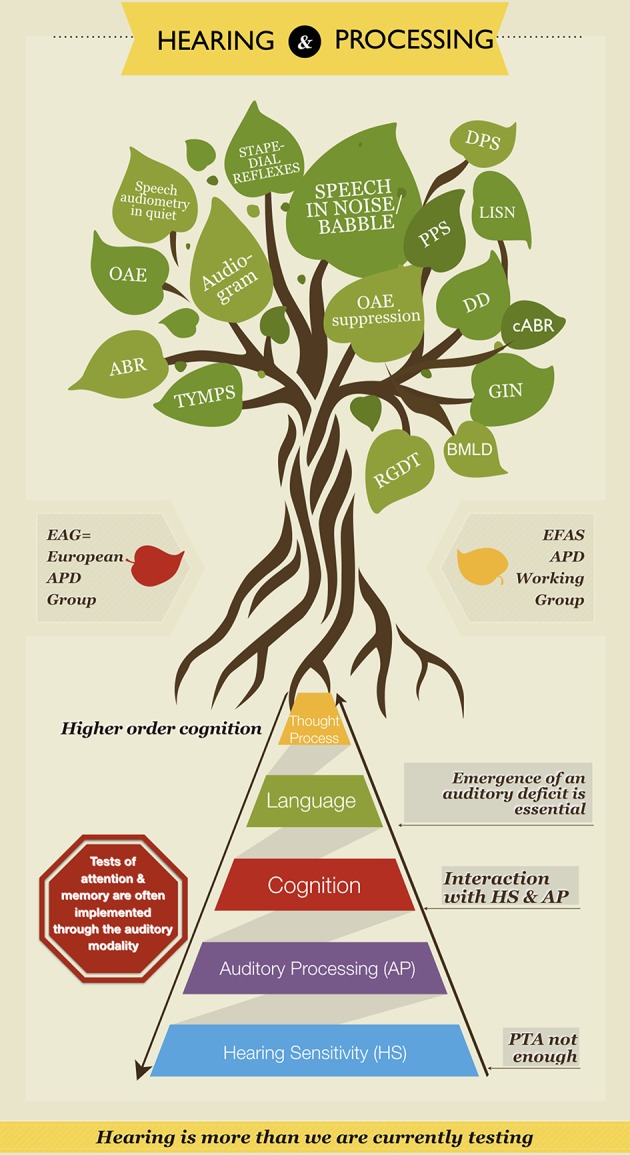
European APD Infographic. The tree at the top is full of the tests of a classical audiology practice on the left side (represented in leaves). These include: the audiogram, pure tone audiometry in quiet, stapedial reflexes, tympanometry (TYMPS), otoacoustic emissions (OAE), and auditory brainstem responses (ABR). The leaves on the right side are tests of auditory processing that are used in specialized APD clinics. These include: speech in noise/babble, pitch pattern sequence (PPS), duration pattern sequence (DPS), dichotic digits (DD), gaps-in-noise (GIN), random gap detection test (RGDT), binaural masking level difference (BMLD), listening in spatialized noise (LISN), otoacoustic emissions suppression (OAE suppression), and complex auditory brainstem response (cABR). On the bottom half of this infographic designed by the European APD Study Group and as part of the European Federation of Audiology Societies (EFAS) APD Working Group the two way pyramid provides a visual of the complex interaction between hearing [hearing sensitivity (HS) + auditory processing (AP)]and cognition. This is an original image, developed by the first author Vasiliki (Vivian) Iliadou.

A recent European APD infographic (Figure 1) was designed to raise awareness of the fact that hearing is more than we are currently testing. Emergence of an auditory deficit poses deleterious effects on language, cognition, learning, and communication. We should not forget that tests of attention and memory are often administered through the auditory modality and may be influenced by a hearing impairment—APD included (23, 24, 31, 32). Auditory processing tests included in the graph are the ones used commonly in practice by European clinicians who run specialty APD clinics. The information in the infographic is based on a questionnaire completed by members of the European APD group of 21 countries.

## Is There Place for New Tests or Approaches to APD?

We certainly believe that APD diagnosis will evolve to include more efficient, reliable, and ecologically valid tests (i.e., tests that reflect everyday hearing situations). One such test may be suppression of otoacoustic emissions using contralateral noise (33, 34). In addition, electrophysiological procedures may be used for APD diagnosis. For example, evidence of pre-attentional auditory discrimination deficit was reported in a recent mismatch negativity study in children diagnosed with APD (35). As science and clinical practices advance, however, it is neither ethical, nor practical to simply reject current clinical expertise as formulated in guidelines of audiology societies and consensus groups around the world. There is no evidence base for doing so and undermining clinical services for millions of patients around the world. Neijenhuis et al. (1) criticize the diagnostic criteria for APD because there is no specification of the number of tests or types of tests that are to be used [although that claim is inconsistent with Weihing et al. (30) and Musiek et al. (31)]. In fact, there is no specific number of tests promulgated for most clinical diagnoses. Diagnosing a disease or a disorder is a process that depends on symptoms, test findings, and patterns identified by the diagnosing clinician. Further, it is an iterative process by which clinical hypothesis are formulated on the basis of the patient's presentation and then confirmed, modified or discarded on the basis of the range of findings and additional information (36). This process cannot be successfully defined or replicated on the basis of a strict set of mathematical rules and criteria: most of the current computer-based diagnostic support systems fail to reach expert clinician diagnostic accuracy levels (36). It should be pointed out that emerging technologies give promising results in modeling out both audiometry (37) and complex auditory perception (38) by means of artificial intelligence and machine learning approaches. At this point in time, however, an experienced clinician is still a more accurate diagnostician than a machine. Emerging technologies augment management and treatment of auditory deficits as well (39, 40).

## Is the Neijenhuis et al. Opinion Article About APD?

We would argue it is not. It focuses on research using non-diagnosed participants bearing the amorphous, non-diagnostic labels “suspected of” or ‘listening difficulties.'

## What Does the Evidence-based Approach to APD Demand?

We must deploy the most efficient (sensitive and specific) available test batteries to diagnose and plan intervention for individuals with APD to minimize the adverse impacts the disorder is causing for communication, education, social integration, and work/livelihoods. APD should be considered within the construct of hearing impairment and should be managed based on diagnosed deficits in central auditory processing.

## Author Contributions

This perspective paper is based on the European APD Consensus Iliadou et al. (5). A first draft was prepared by VI. CK-H, D-EB, HG, MP, GC, HT-V, TS, and FM made substantial contributions/edits/modifications to the conception of this paper and were involved in drafting the work and/or revising it critically for important intellectual content. Final approval of the version to be published was provided by all listed authors. All listed authors agree to be accountable for all aspects of the work in ensuring that questions related to the accuracy or integrity of any part of the work are appropriately investigated and resolved.

### Conflict of Interest

The authors declare that the research was conducted in the absence of any commercial or financial relationships that could be construed as a potential conflict of interest.

## References

[1] Neijenhuis K Campbell NG Cromb M Luinge MR Moore DR Rosen S. An evidence-based perspective on misconceptions regarding pediatric auditory processing disorder. Front Neurol. (2019) 10:287. 10.3389/fneur.2019.00287 30972015 PMC6443922

[2] Sackett DL Strauss DE Richardson WS Rosenberg W Evidence-Based Medicine: How to Practice and Teach EBM. 2nd ed Edinburgh: Churchill Livingstone (2000).

[3] Haynes RB Devereaux PJ Guyatt GH Clinical expertise in the era of evidence-based medicine and patient choice. BMJ Evid Based Med. (2002) 7:36–8. 10.1136/ebm.7.2.36 12749371

[4] Sedgwick P (2013). Generalisation and extrapolation of study results. BMJ. 346:f3022 10.1136/bmj.f3022

[5] Iliadou V Ptok M Grech H Pedersen ER Brechmann A Deggouj N. A European perspective on auditory processing disorder-current knowledge and future research focus. Front. Neurol. (2017) 8:622. 10.3389/fneur.2017.00622 29209272 PMC5702335

[6] Heine C O'Halloran R. Central auditory processing disorder: a systematic search and evaluation of clinical practice guidelines. J Eval Clin Pract. (2015) 21:988–94. 10.1111/jep.12494 26687837

[7] Siering U Eikermann M Hausner E Hoffmann-Eßer W Neugebauer EA. Appraisal tools for clinical practice guidelines: a systematic review. PLoS ONE. (2013) 8:e82915. 10.1371/journal.pone.0082915 24349397 PMC3857289

[8] de Wit E Visser-Bochane MI Steenbergen B van Dijk P van der Schans CP Luinge MR. Characteristics of auditory processing disorders: a systematic review. J Speech Lang Hear Res. (2016) 59:384–413. 10.1044/2015_JSLHR-H-15-0118 27082630

[9] Tomlin D Dillon H Sharma M Rance G. The impact of auditory processing and cognitive abilities in children. Ear Hear. (2015) 36:527–42. 10.1097/AUD.0000000000000172 25951047

[10] Chermak GD Musiek FE Weihing J Beyond controversies: the science behind central auditory processing disorder. Hear Rev. (2017) 24:20–4. Available online at: http://www.hearingreview.com/2017/05/beyond-controversies-science-behind-central-auditory-processing-disorder/

[11] Brenneman L Cash E Chermak GD Guenette L Masters G Musiek FE The relationship between central auditory processing, language, and cognition in children being evaluated for central auditory processing disorder (CAPD). J Am Acad Audiol. (2017) 28:758–69. 10.3766/jaaa.16119 28906246

[12] Rosen S Cohen M Vanniasegaram I. Auditory and cognitive abilities of children suspected of auditory processing disorder (APD). Int J Pediatr Otorhinolaryngol. (2010) 74:594–600. 10.1016/j.ijporl.2010.02.021 20347161

[13] Sharma M Purdy SC Kelly AS. Comorbidity of auditory processing, language, and reading disorders. J Speech Lang Hear Res. (2009) 52:706–22. 10.1044/1092-4388(2008/07-0226) 19064904

[14] American Speech-Language-Hearing Association Central Auditory Processing Disorders. (2005). Available online at: www.asha.org/POLICY/TR2005-00043/ (accessed March 20, 2019).

[15] American Academy of Audiology (AAA) Practical Guidelines for the Diagnosis, Treatment, and Management of Children and Adults with Central Auditory Processing Disorder (CAPD). (2010). Available online at: www.audiology-web.s3.amazonaws.com/migrated/CAPD%20Guidelines%208-2010.pdf_539952af956c79.73897613.pdf

[16] Stavrinos G Iliadou VV Edwards L Sirimanna T Bamiou DE. The relationship between types of attention and auditory processing skills: reconsidering auditory processing disorder diagnosis. Front Psychol. (2018) 9:34. 10.3389/fpsyg.2018.00034 29441033 PMC5797617

[17] Gyldenkærne P Dillon H Sharma M Purdy SC. Attend to this: the relationship between auditory processing disorders and attention deficits. J Am Acad Audiol. (2014) 25:676–87. 10.3766/jaaa.25.7.6 25365370

[18] Weihing J Chermak GD Musiek FE. Auditory training for central auditory processing disorder. Semin Hear. (2015) 36:199–215. 10.1055/s-0035-1564458 27587909 PMC4910543

[19] Banai K Sabin AT Wright BA. Separable developmental trajectories for the abilities to detect auditory amplitude and frequency modulation. Hear Res. (2011) 280:219–27. 10.1016/j.heares.2011.05.019 21664958 PMC3179807

[20] Dawes P Bishop DVM. Maturation of visual and auditory temporal processing in school-aged children. J Speech Lang Hear Res. (2008) 51:1002–15. 10.1044/1092-4388(2008/073) 18658067

[21] Moore DR Cowan JA Riley A Edmondson-Jones AM Ferguson MA. Development of auditory processing in 6- to 11-yr-old children. Ear Hear. (2011) 32:269–85. 10.1097/AUD.0b013e318201c468 21233712

[22] Sidiras C Iliadou VV Nimatoudis I Grube M Griffiths T Bamiou D-E. Deficits in auditory rhythm perception in children with auditory processing disorder are unrelated to attention. Front Neurosci. (2019) 13:953. 10.3389/fnins.2019.00953 31551701 PMC6743378

[23] Iliadou V Moschopoulos N Sidiras C Eleftheriadou A Nimatoudis I Over-diagnosis of cognitive deficits in psychiatric patients may be the result of not controlling for hearing sensitivity and auditory processing. Psychiatry Clin Neurosci. (2018) 72:742 10.1111/pcn.12768 29999211

[24] Iliadou V Moschopoulos N Sidiras C Eleftheriadou A Nimatoudis I Inaccurately measured poorer cognition as a result of an auditory deficit. J Psychol Psychiatry. (2018) 2 10.15761/JPP.1000117

[25] American Psychiatric Association Diagnostic and Statistical Manual of Mental Disorders. 5th ed Arlington, VA: American Psychiatric Publishing (2013).

[26] Deutsche Gesellschaft für Phoniatrie und Pädaudiologie (German Society of Phoniatrics and Pediatric Audiology) Auditory Processing and Perception Disorder (Auditive Verarbeitungs- und Wahrnehmungsstörungen, AVWS). (2015). Available online at: http://www.awmf.org/leitlinien/detail/ll/049-012.html

[27] Ptok M Kiese-Himmel C Nickisch A Guideline: auditory processing and perception disorders: definition. guideline of the german society of phoniatrics and pediatric audiology. HNO. (2019) 67:8–14. 10.1007/s00106-018-0598-y 30523378

[28] Kiese-Himmel C Nickisch A. Diagnostic accuracy of a test set to classify children with Auditory Processing Disorders (APD). Laryngorhinootology. (2015) 94:373–7. 10.1055/s-0034-1387766 25429641

[29] Musiek FE Chermak GD Weihing J Zappulla M Nagle S. Diagnostic accuracy of established central auditory processing test batteries in patients with documented brain lesions. J Am Acad Audiol. (2011) 22:342–58. 10.3766/jaaa.22.6.4 21864472

[30] Weihing J Guenette L Chermak GD Brown M Ceruti J Fitzgerald K Characteristics of pediatric performance on a test battery commonly used in the diagnosis of central auditory processing disorder (CAPD). J Am Acad Audiol. (2015) 26:652–69. 10.3766/jaaa.14108 26218054

[31] Lin FR Albert M Editorial: Hearing loss and dementia – Who's listening? Aging Ment Health. (2014) 18:671–73. 10.1080/13607863.2014.915924 24875093 PMC4075051

[32] Warren JD Bamiou DE. Prevention of dementia by targeting risk factors. Lancet. (2018) 391:1575. 10.1016/S0140-6736(18)30579-8 29695344

[33] Iliadou VV Weihing J Chermak GD Bamiou DE. Otoacoustic emission suppression in children diagnosed with central auditory processing disorder and speech in noise perception deficits. Int J Pediatr Otorhinolaryngol. (2018) 111:39–46. 10.1016/j.ijporl.2018.05.027 29958612

[34] Guinan JJ Jr. Olivocochlear efferents: their action, effects, measurement and uses, and the impact of the new conception of cochlear mechanical responses. Hear Res. (2018) 362:38–47. 10.1016/j.heares.2017.12.012 29291948 PMC5911200

[35] Rocha-Muniz CN Lopes DMB Schochat E. Mismatch negativity in children with specific language impairment and auditory processing disorder. Braz J Otorhinolaryngol. (2015) 81:408–15. 10.1016/j.bjorl.2014.08.022 26142650 PMC9442763

[36] Kohn MA. Understanding evidence-based diagnosis. Diagnosis. (2014) 1:39–42. 10.1515/dx-2013-0003 29539978

[37] Barbour DL Howard RT Song XD Metzger N Sukesan KA DiLorenzo JC. Online machine learning audiometry. Ear Hear. 40, 918–26. 10.1097/AUD.0000000000000669 30358656 PMC6476703

[38] Gallun FJ Seitz A Eddins DA Molis MR Stavropoulos T Jakien KM. Development and validation of portable automated rapid testing (PART) measures for auditory research. Proc Meet Acoust. (2018) 33:050002. 10.1121/2.0000878 30627315 PMC6322842

[39] Olson AD. Options for auditory training for adults with hearing loss. Semin Hear. (2015) 36, 284–95. 10.1055/s-0035-1564461 27587915 PMC4910545

[40] Keith WJ Purdy SC Baily MR Kay FM New Zealand Guidelines on Auditory Processing Disorder. New Zealand Audiological Society (2019). Available online at: https://www.audiology.org.nz/

